# Forest biomass carbon stocks and variation in Tibet’s carbon-dense forests from 2001 to 2050

**DOI:** 10.1038/srep34687

**Published:** 2016-10-05

**Authors:** Xiangyang Sun, Genxu Wang, Mei Huang, Ruiying Chang, Fei Ran

**Affiliations:** 1 Institute of Mountain Hazards and Environment, Chinese Academy of Sciences, Chengdu Sichuan 610041, China; 2 Institute of Geographic Sciences and Natural Resources Research, Chinese Academy of Sciences, Beijing 100101, China.

## Abstract

Tibet’s forests, in contrast to China’s other forests, are characterized by primary forests, high carbon (C) density and less anthropogenic disturbance, and they function as an important carbon pool in China. Using the biomass C density data from 413 forest inventory sites and a spatial forest age map, we developed an allometric equation for the forest biomass C density and forest age to assess the spatial biomass C stocks and variation in Tibet’s forests from 2001 to 2050. The results indicated that the forest biomass C stock would increase from 831.1 Tg C in 2001 to 969.4 Tg C in 2050, with a net C gain of 3.6 Tg C yr^−1^ between 2001 and 2010 and a decrease of 1.9 Tg C yr^−1^ between 2040 and 2050. Carbon tends to allocate more in the roots of fir forests and less in the roots of spruce and pine forests with increasing stand age. The increase of the biomass carbon pool does not promote significant augmentation of the soil carbon pool. Our findings suggest that Tibet’s mature forests will remain a persistent C sink until 2050. However, afforestation or reforestation, especially with the larger carbon sink potential forest types, such as fir and spruce, should be carried out to maintain the high C sink capacity.

Terrestrial vegetation plays an important role in the global carbon cycle and in alleviating atmospheric CO_2_ elevation^1^. The global terrestrial ecosystem’s gross primary production is 123 ± 8 petagrams of carbon per year (Pg C year^−1^), and forests account for 80% of this production^2^. In addition, forests have political value in terms of the Reducing Emissions from Deforestation and Degradation (REDD) scheme^3^. Currently, this global carbon sink absorbs 30% of fossil fuel CO_2_ emissions^4^,^5^. Deforestation and forest degradation account for 12–20% of global anthropogenic greenhouse gas emissions, and both processes can affect the efficiency of forests to remove carbon from the atmosphere^6^. If the global forest carbon sink is reduced or eliminated, then global efforts to mitigate climate change will require additional emission reductions^7^. From a climate change mitigation perspective, the key management issues are determining the best methods to avoid carbon emissions and to maximize long-term carbon storage in forests^8^,^9^,^10^. Therefore, it is important to evaluate the biomass carbon stock and sequestration potential for emission-reduction policy making^11^.

In past decades, primary old forests have been regarded as unimportant in mitigating the climate change problem because they were considered carbon neutral and less efficient as carbon sinks than young forests. However, recent findings have demonstrated that very old forests do not reach equilibrium between carbon assimilation and respiration and can still increase their carbon biomass^12^,^13^ and enhance the soil carbon stock^14^,^15^. Old forests are often biomass carbon-dense forests and have less anthropogenic and natural disturbances, cool temperatures and moderate precipitation, all of which contribute to the accumulation of biomass carbon^13^,^16^,^17^. The difference between the current biomass carbon stock and the carbon stock in old forests was used to predict the carbon sink potential on the regional and global scales^18^. However, using this method to evaluate the carbon sink potential is difficult because the selection of old forests is incomplete due to the lack of sample data^19^.

Current estimates of the carbon stock are usually based on the upscaling of often sparse forestry inventory data to national estimates^4^,^5^,^20^; however, these estimates cannot offer spatial details of a forest’s biomass and its variations. Conversely, the integration of forest inventory data with remote sensing data or satellite passive microwave imagery has been used to investigate the spatial distribution of forest biomass and its variations^19^,^21^. However, these methods only provide robust forest types and limited temporal frequency and are unable to predict the carbon stock potential. In forest management for climate mitigation, it is important to determine the spatial distribution of the biomass carbon within biomes and their carbon sink potential^16^,^19^,^21^.

The difference between the carbon carrying capacity and the current carbon stock allows estimation of the carbon potential of an ecosystem^16^,^18^. High-biomass forests have been estimated to be a small carbon source; however, there are high uncertainties associated with this estimation that may be caused by the lack of carbon-dense samples^19^. Xu *et al*.^22^ estimated the potential biomass carbon stocks of China’s forests for 2000 and 2050 based on the relationship of the biomass and forest age group, without considering forest mortality and harvest^22^. Hu *et al*.^23^ re-estimated the potential biomass carbon stocks using stage-classified matrix models based on the national inventory data and demonstrated that the carbon density and carbon sinks were overestimated by Xu *et al*.^22^,^23^. However, no real forest ages or spatial distributions of biomass carbon within the biomes provided were determined, and the national model parameters, in particular, may not be suitable for carbon-dense forests. Because the national forestry inventories may not be suitable for estimating carbon stocks at the national and biome scale, these results have been questioned^24^,^25^. The most reliable biomass carbon data are from field measurements at sites for a given forest type and condition^16^.

In the majority of China, forests are characterized by a young age, low carbon density and large plantation area^23^,^26^,^27^. However, Tibet’s forests are characterized by an old age, high carbon density and small plantation area^19^,^21^,^28^. Additionally, Tibet’s forests account for 39.2% of the forest area on the Qinghai-Tibetan Plateau and are the core carbon sinks in China, playing an important role in China’s carbon sinks^29^. Although the national forest inventory data have shown that these forests are a large carbon sequestration area and carbon sink in China^23^, there is a lack of knowledge of the spatial distribution and carbon sink potential of the carbon-dense forests in Tibet. This presents a significant problem for forest management efforts to mitigate climate warming on both the regional and national scale. Forests are a major part of Tibet’s terrestrial ecosystem and play a special role in terms of global warming and safeguarding the ecological balance. Here, we extend the field measurement data with the spatial distribution of forest types and stand age for two reasons: 1) to derive the spatial distribution of the forest biomass carbon density and 2) to predict the variation of the carbon sink until 2050. We then present a forest management strategy for carbon-dense forests.

## Materials and Methods

### Study area

**Figure 1:**
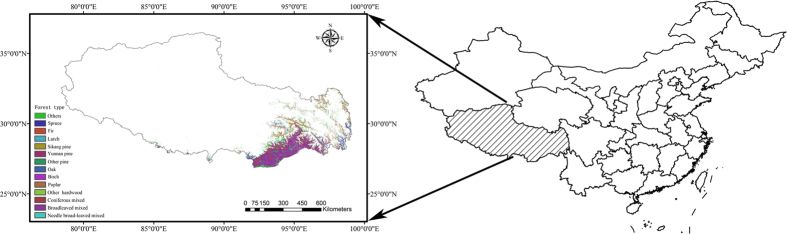
Location of the study area and distribution of the forest types. Maps were generated using ArcGIS 9.3 (www.esri.com/software/arcgis).

The Xizang (Tibet) Autonomous Region is located in southwestern China and occupies an area of 122.84 × 10^4^ km^2^. The Tibetan Plateau plays an important role in the climate, hydrology and biology of China. This study was conducted in the southeastern and southern mountainous areas of Tibet, including Nyingchi, Qamdo, Shigatse, Shannan and Matuo, which are adjacent to each other and are located on the southeastern Tibetan Plateau (Fig. 1). The climate in Tibet is variable, with precipitation ranging from 397 mm to 1910 mm and mean annual air temperature ranging from 1.7 °C to 15.5 °C (data from WorldClim data set, http://www.worldclim.org/current). This is the main area of Tibetan virgin forests and the principal part of the southwestern Chinese national forests. The forest area is 8.52 × 10^4^ km^2^, which is 6.9% of the total area in Tibet^30^. The forests have special tree species and intact vertical distributions due to the unique topography of Tibet. The main forest types are subalpine dark coniferous forest, temperate mountain pine forest, temperate sclerophyllous evergreen robur forest, mountain deciduous broad-leaved forest and mountain cypress forest^31^. The area of spruce, fir and broadleaved mixed forest were larger than other forest types in Tibet (Fig. S1). The soil types vary in the different forest types and vertical zones, including loamy Humic Cambisols, Haplic Luvisols, Albic Luvisols and Luvisols^30^,^32^.

### Forest inventory and sample measurement

Forest inventory sites were selected based on the distribution of forest types, which were acquired from a land cover map of Tibet and field surveys. In total, 413 forest plots were selected for forest inventory during 2011 and 2013. The field sampling protocol was followed by the distribution weight, relative distribution of the forest community and the forest management regime^33^. In general, the sampled forest sites were evenly distributed for each forest type. At each site, three inventory forest plots (50 m by 20 m) with the same dominant species were established, and the distance between repeated plots was more than 300 m. The altitude, longitude and elevation of each plot were recorded using a GPS navigator, and the aspect and slope were measured using a compass. Within each of the inventory plots, three quadrats (10 m × 10 m) were established to sample soil and trees. In each plot, the number of trees was measured for all of the live trees with a diameter at breast height (DBH) ≥ 5 cm and with a height ≥ 2 m. The DBH was measured 1.30 m aboveground. Tree heights were measured using an ultrasonic altimeter (Vertex IV, Haglöf, Sweden). Forest age was determined using sampled tree ring cores and represent the average age of the trees on the sample plot^34^; we divided the DBH to 5 classification, and selected 4 to 5 trees randomly to measure sample tree ring cores for each classification. Three trees with small, medium and large DBHs within each quadrat were selected. Leaves, branches with bark, stems (including sapwood, heartwood and bark) and roots (including fine (diameter < 2 mm), medium (2 mm < diameter < 30 mm), and coarse (diameter > 30 mm)) of each tree were collected separately. Three repetitions of the same type of samples were mixed together to form one sample. All the tree samples were sealed in plastic bags. In the laboratory, the samples were oven-dried at 60 °C to a constant weight. Drug pulverizers were used to grind the samples to a powder to pass through a 0.15-mm mesh sieve. The carbon and nitrogen concentrations of all the tree samples were determined using a C/N analyzer (Multi-N/C 2100, Analytik Jena AG, Germany).

The biomass data for the forests were obtained using the sample tree determination method during the inventory period. The DBHs were divided into seven classes depending on the forest type. In general, each forest type was distributed along an elevation gradient. Therefore, the felled sample trees were also sampled from different altitudinal zones at 200 m elevation intervals. In each altitudinal zone, 2-3 trees were collected for each of the DBH classes, and a total of 14–21 trees were felled. The total number of sample trees used to calculate the biomass was based on the distribution range of the elevation gradient. The branches, twigs, leaves, roots and stems were collected separately. For convenience of transportation and analysis, only 1/10 of the equally divided parts of the samples were collected. The fresh weights in each category were measured and then oven-dried in the lab at 60 °C to a constant weight. The same sample trees were used to develop site-specific allometric equations to estimate the biomass of the stems, leaves, branches and roots. The equations were valid for all the forest types in this study.

In each plot, three quadrats (1 m × 1 m) were randomly selected to sample the organic and mineral soil based on the method described by Hoover^35^. After collecting the organic layer, the mineral soil was collected from a 0 cm to 50 cm depth with an interval of 10 cm. Similar to the tree samples, the three soil samples were collected repeatedly from the three profiles in each plot and were mixed to form one sample. Meanwhile, the soil bulk density samples were collected via core drills at each interval layer in each quadrat. The coarse fraction, coarse roots, litter detritus and fine roots were removed using a mesh sieve and glass bar. The soil samples were oven-dried at 60 °C and then ground to pass through a 0.15 mm mesh sieve to analyze the soil total carbon (SC) and total nitrogen (TN) concentrations (Vario Macro cube, Elementar, German).

### Carbon storage and carbon density estimates

Species-specific allometric equations were developed using the tree height and DBH as independent variables. A least-squares regression model was used to determine the coefficients of height and DBH in the allometric equations. The correction factor, CF, the adaptability index, FI, and the standard error were used to correct for systematic bias^30^. Using the field sampling data of the stems, branches, leaves and roots from different forest types, allometric equations for the live biomass were developed separately. The carbon storage could then be calculated as the biomass multiplied by the carbon concentration of each component of the leaves, braches, stems and roots. The total forest carbon storage was calculated as the sum of the four components. The carbon density was derived from the inventory total carbon storage divided by the inventory area.

### Map of forest age

Wang *et al*.^36^ compiled a forest stand age map of China in 2001 based on the national forest inventory data between 1989 and 1993^36^,^37^. The forest age was the sum of the mean age and the standard deviation in each pixel. The standard deviation was determined according to polygon data in each province. The hypothesis was that the forest age was distributed normally in each forest polygon. The forest age map was based on a 1:4 million vegetation map from Wang *et al*.^36^, the forest area was larger than the 1:1 million map^36^, and the distributions of forest types were different between the two scaled maps. The forest age distributed complex. Therefore, the coarse resolution map of forest age was re-sampled by particle filter method with nonlinear models to get the finer map^38^. Then, the forest ages were increased by 10 to obtain a forest age map for 2010. The forest ages of the 413 inventory plots of different forest types were integrated into the new forest age map to improve the accuracy in Tibet. This map, with 2010 as the base year, enables the analysis of relationship between carbon density and forest age. The classification of forest ages (young, middle-age, pre-mature, mature and over-mature) for different forest types was based on Xu *et al*.^22^ and Hu *et al*.^23^.

### Relationship between carbon density and forest age

In general, the inventory sites experienced slight and moderate disturbances from human activities; severe disturbances were rare. Both forest regeneration and mortality occurred naturally with increasing forest age; therefore, we concluded that the inventory data of different forest ages also represented the natural succession process. Based on the inventory data of the forest age and carbon density in live biomass, we used logistic curves to fit the relationship between the carbon density and the forest age for each forest type.

The equation of the curve was as follows:


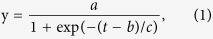


where *y* is the carbon density, *t* is the forest age and *a*, *b* and *c* are constants for a specific forest type. Xu *et al*.^22^ successfully used this equation to estimate and predict the biomass in China’s forests^22^. However, we found that Eq. 1 was not suitable for all forest types, such as *Pine densata*, *Populus* and mixed broadleaf-conifer forest and that another pattern of the carbon density-age relationship provided a better fit (with larger R^2^ values). This equation was as follows:


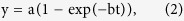


where *a* and *b* are constants for specific forest types.

We assume that the forest area in the national inventory from 2009 to 2011 represents the forest distribution in 2010 and that neither clear-cuts nor die-offs occur prior to 2050. Although the total area and coverage of forests will increase dramatically by 2050 in China (a projected forest coverage increase of 20.1% in 2005 to 28.4% in 2050), according to China’s forestry development goals^23^,^39^, the area of afforestation is generally negligible in Tibet. Therefore, we calculated Tibet’s total carbon storage in its existing natural forest for a particular year using Eqs (1) and (2) to map the forest age and forest area in 2010.

## Results

### Spatial patterns of Tibet’s forest stand age

**Figure 2:**
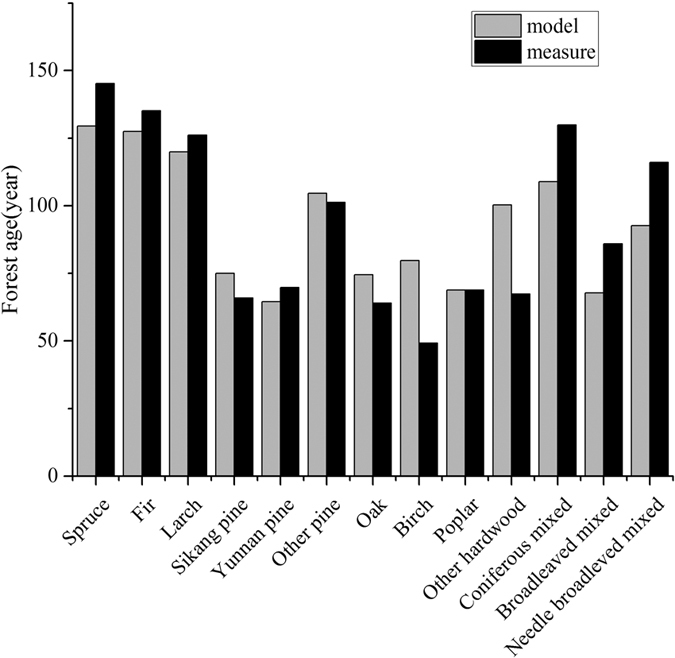
The comparisons of modeled and measured mean forest age. The model value was based on the relationship between forest age and NPP with the forest inventory data^36^. The measure value was from the forest inventory data in 2011.

In general, the estimated average forest ages for each forest type are in good agreement with the field inventory data in 2011 (Fig. 2). However, the biases for birch and other hardwoods are 30 years and 32 years higher than the forest inventory data, respectively. Forested areas in these two forest types account for 1.8% and 2.7% of the Tibetan total, and their stand volumes account for 0.7% and 2.4% of the total. Therefore, these errors are acceptable in this study.

**Figure 3:**
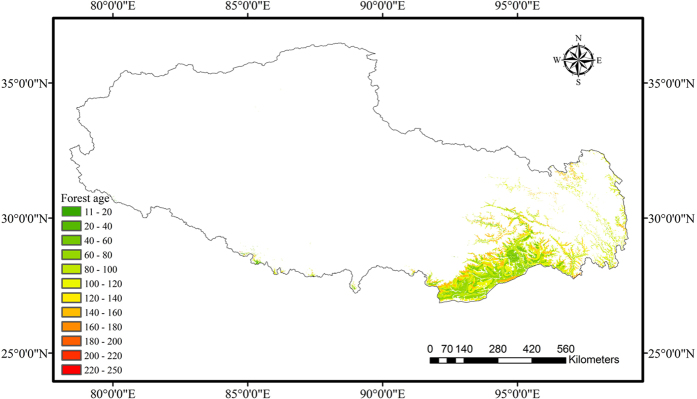
Distribution of forest age in Tibet in 2010. Map was generated using ArcGIS 9.3 (www.esri.com/software/arcgis).

The estimated forest ages range from 11 years to 250 years. The distribution of the estimated forest ages shows high spatial heterogeneity, which reflects the differences in topographical conditions and forest types (Fig. 3). By statistical analysis of the 0.01 × 0.01 degree resolution grid map of forest stand age, the age classes 11–20, 21–40, 41–60, 61–80, 81–100, 101–120, 121–140, 141–160, 161–180 and more than 181 occupied 0.15%, 0.19%, 9.2%, 18.2%, 27.5%, 25.8%, 4.2%, 14.6%, 0.1% and 0.1% of the total Tibetan forest area, respectively. The map suggests that the middle-aged, pre-mature, mature and over-mature trees currently compose the majority of the Tibetan forest. The definitions of forest age groups were taken from Xu *et al*.^22^ for the different forest types. These results were the same as those of the forest inventory data of Tibet in 2011, which showed that young-aged stands only occupy 7.24% of the total forest area (Table 1).

### Relationship of carbon density and forest age

The functions of carbon density and forest age were established based on the 413 field sites. For each forest type, the forest age was varied from young to overly mature to confirm the accuracy of the equations, especially for mature and overly mature forests. The patterns of the carbon density-age equation were primarily determined by the correlation coefficient (*R*^*2*^), with an *R*^*2*^ of more than 0.7 (p < 0.05). The majority of the forest types, such as spruce, fir, larch, pine, oak and Birch, were best fit with Eq. (1). However, Eq. (2) was more appropriate for other forest types, such as sikang pine, poplar and needle- and broad-leaf mixed forests. The parameters of the equations relating carbon density and forest age for all of the forest types are shown in Table 2. Using these equations, we can estimate the carbon density and carbon stock with greater accuracy.

### Carbon stocks and carbon sink potential

**Figure 4:**
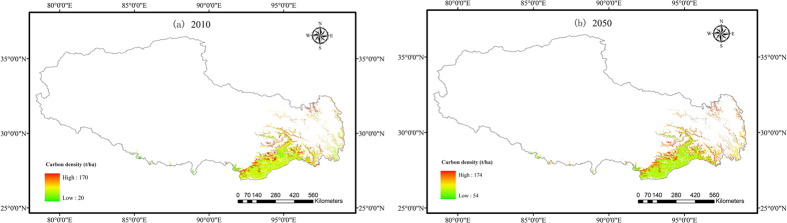
The spatial distribution of carbon density (t/ha) in 2010 (**a**) and 2050 (**b**) (stem + branches + roots + foliage). Maps were generated using ArcGIS 9.3 (www.esri.com/software/arcgis).

We estimated the carbon stocks for both the aboveground and belowground live biomass of the Tibetan forests from 2001 to 2050 using the carbon density-age equations. The total carbon stock was 866.8 Tg C in 2010 (Table 3), and the carbon density varied between 20 t/ha and 170 t/ha (Fig. 4a). Fir had the largest mean carbon density of 147.1 t/ha, whereas poplar had the lowest mean carbon density of 31.8 t/ha. The spatial distributions of the carbon stocks were influenced by both forest age and forest type. The carbon stocks were lower in the southwest forest areas because low carbon density forests dominate those areas.

**Figure 5:**
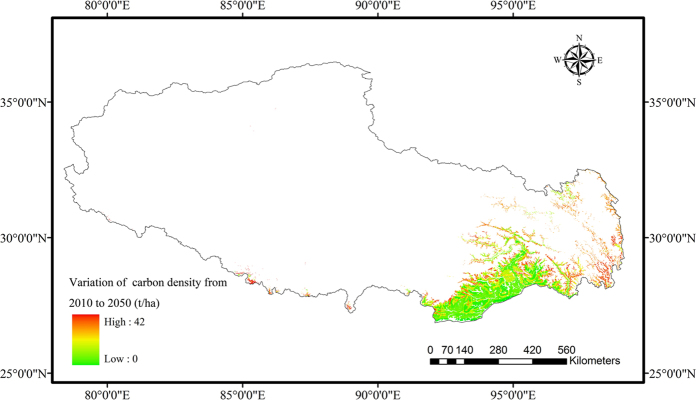
Variation of carbon density from 2010 to 2050 in Tibet. Map was generated using ArcGIS 9.3 (www.esri.com/software/arcgis).

The total forest carbon stock will increase by 16.6% from 831.1 Tg C in 2001 to 969.4 Tg C in 2050. The mean carbon density will increase by 16.0% from 99.1 t/ha in 2001 to 115.6 t/ha in 2050 (Table 3). The spatial distribution of the carbon density will vary between 54 t/ha and 174 t/ha in 2050 (Fig. 4b). The net variation of carbon density will be from 0 t/ha to 42 t/ha, with low variation in the southwest forest areas (Fig. 5). This implies that forests with low carbon density sequester carbon more efficiently, while high carbon density areas continue to sequester carbon but with much lower efficiency. The variation in the carbon stocks is also determined by the forest type. The effect of both forest type and forest age determined the spatial pattern of the carbon stock variation.

**Figure 6:**
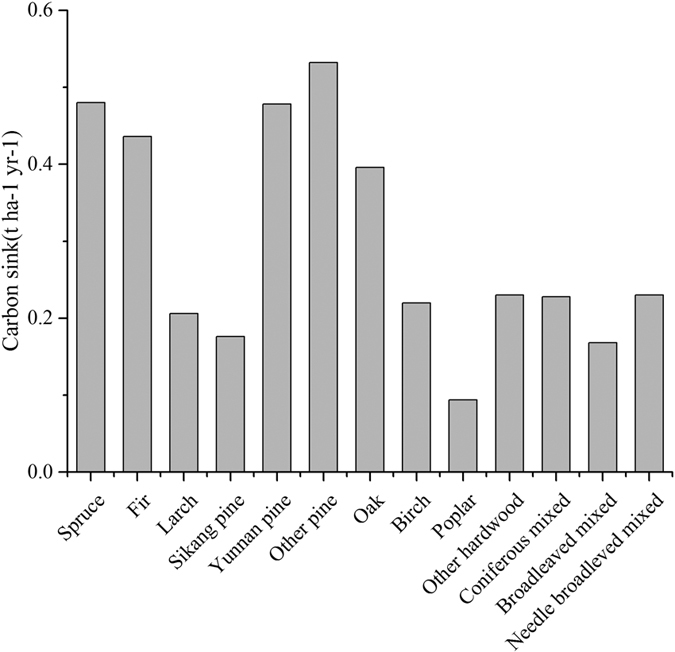
Annual carbon sink for different forest types between 2001 and 2050 in Tibet.

The increase in both carbon density and carbon stock suggests that the Tibetan forests will function as a carbon sink over the next 40 years. However, the annual forest biomass carbon sink will decrease by 47.2% from 3.6 Tg C yr^−1^ between 2001 and 2010 to 1.9 Tg C yr^−1^ between 2040 and 2050. The age of spruce and fir forests are older, but still provide a greater carbon sink (Fig. 6). Yunanan pine, oak and other Pine have higher carbon sinks but generally younger forest ages (Fig. 6). In general, other forest types have lower carbon sinks. The total carbon stock will increase annually from 2001 to 2050 at a mean rate of 2.8 Tg C yr^−1^.

## Discussion

### Uncertainty analysis

This study provides a comprehensive assessment of forest biomass carbon stock in the Tibetan subalpine forest area. Due to the extreme climatic conditions and forest inventory process, national forest inventory data can only provide the forest type, forest area, the classification of forest ages and stem volume for China’s national forest inventory, which have been used in previous national-scale forest biomass and carbon stock analyses. Other method, such as satellite data can be used to generate the spatial pattern of forest biomass, but MODIS spectral bands could only explain 75% of the biomass, and the lack of enough high biomass samples could introduce more uncertainty^19^. The use of different methods to calculate the forest biomass carbon stocks from stem volume can generate variable results^40^. Fang *et al*.^4^ demonstrated that the most important uncertainties resulted from the forest inventory data and biomass expansion factor (BEF) method, especially at the province scale^4^. In contrast, the higher sampling density and the separate analysis of branches, twigs, leaves and roots in this study will help to reduce the uncertainties in regional forest carbon stock estimates in Tibet. Diameter is the most common predictor in all biomass allometric models^41^. Some studies found that tree height can improve the statistically significance of models^42^, but tree height was rarely used. The reason is that tree height was not easy to estimate accurately with the influence of terrain and dense forest, allometric models adding tree height may introduce propagated variance due to the variance caused by tree height measurements^43^. Therefore, DBH-only equations were applied for total biomass estimation to minimize the uncertainty of parameters (Table S1). The carbon content of each component was different within and between different forest types (Table S2), the calculation with different carbon content also can reduce the error of carbon density^41^. However, several factors may still introduce uncertainties in our study.

We used a constant forest distribution map, which is unable to account for the dynamic response of forest areas during different periods, especially as the planted forest area increases. The planted forests only account for a very small fraction of the carbon stock in Tibet (0.04 Tg C from 1998–2010 vs. 866.8 Tg C in 2010)^28^. Therefore, we concluded that the planted forest did not play a significant role in determining the spatial and temporal pattern of the carbon stock in Tibet. Pan *et al*.^5^ compiled a forest age map of North America by combining forest inventory data and historical fire data^5^. However, fire events were not included in this study for the forest age map because we had no fire data, and fire events are also not frequent due to strict management. Zhang *et al*.^44^ generated a forest stand age map in China based on forest height data, and the results showed that the forests in Tibet were older than in other areas in China^44^. However, the estimated oldest forests also included larger uncertainties because of the sparse data^44^. The mean estimated forest age was in accordance with the forest inventory data for each forest type (Fig. 2). Therefore, the forest age map derived by forestry inventory and field measurements represents the situation of forest growth in Tibet.

Forest age, nitrogen deposition, climate change, atmospheric CO_2_ and other environmental factors contribute to variation in the carbon density^19^,^45^,^46^. However, the contributions of these effects cannot be isolated and quantified. Global temperature and air CO_2_ concentration have continued to increase since the industrial revolution, and nitrogen deposition has increased due to human activity^47^. The relationships between carbon density and forest age include the variation of these environmental factors because the promotion or restriction of environmental factors on forest growth and carbon sequestration also varies with forest type and stand age.

### Carbon stock and carbon density

The carbon stock and carbon density were 793.9 Tg C and 97.3 Mg C ha^−1^, respectively, from 1994 to 1998 in Tibet^26^, which were slightly lower than those estimated for 2001 in this study (Table 3). The carbon stock in Tibet was lower than that in Inner Mongolia, Heilongjiang, Sichuan and Yunnan Provinces in China; however, the carbon density was nearly twice that in these four provinces^26^. The carbon stock and carbon density of existing forests were 7385 Tg C and 51.7 Mg C ha^−1^, respectively, in China in 2010^22^. Although the forest area of Tibet only accounts for 5.4% of the total natural forest area in China^23^ (8.52 × 10^6^ vs. 15.86 × 10^8^ ha), its carbon stock accounts for 11.7% of China’s total forest area (866.8 Tg C in Tibet in 2010). These comparisons highlight that the Tibetan forest plays an important role in China’s terrestrial carbon storage and carbon cycle.

The mean carbon density of live biomass in Tibet is lower than that in most carbon-dense forest sites in the world^13^,^16^. However, there were 11 investigated sites of spruce, oak and cypress forests with carbon densities greater than 520 t/ha. The mean carbon density was 43 t/ha between 1995 and 1999 in the northern forests^48^, 68.7 t/ha in North America, 60.5 t/ha in Europe and 42.0 t/ha in Asia in 2007^5^ and 45.3 t/ha in North America, 60.8 t/ha in Europe and 43.6 t/ha in Asia based on the growing stock volume data in 2010^20^, which are lower than those in Tibet. This highlights that the carbon density of Tibetan forest was also significance around the world.

The reason for the high biomass carbon densities in Tibet may be that the relatively cool temperature and moderately high precipitation induce fast growth but slow decomposition^16^,^17^,^49^. Older forests are often multi-aged and multilayered and have experienced minimal anthropogenic disturbances. In Tibet, the sparse traffic network, sparse population and natural forest protection result in minimal anthropogenic disturbances in the natural forests^4^. Overall, forests grow with faster growth rates, longer tree longevity and slower corrosion rates in most areas of Tibet than in other areas in China^31^. The unique geography of the Qinghai-Tibetan Plateau causes stronger solar radiation than at similar latitudes. In addition, the relatively low nighttime air temperature and cold and dry climate conditions in the winter cause the respiration to be relatively low^50^. Therefore, the forests can continue to accumulate carbon with forest age^45^, despite the decreasing growth efficiency^13^.

**Figure 7:**
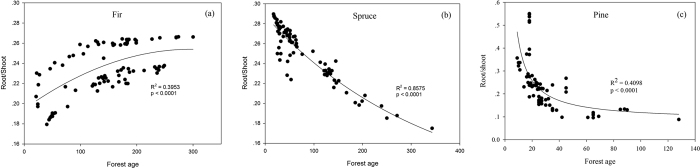
Fitted relationships between root/shoot ratio and forest age for Fir (**a**), Spruce (**b**) and Pine (**c**) respectively using the inventory data.

Although the total carbon storage increased with age, the carbon allocation aboveground and belowground with forest age was different among the forest types, which influenced the patterns of ecosystem carbon accumulation. The root/shoot ratio (defined as the root carbon divided by the shoot carbon, with ‘root’ referring to carbon in the root and ‘shoot’ referring to carbon in the leaves, branches and stems) represents an accurate estimation of carbon allocation aboveground and belowground^30^,^51^. The results showed that the root/shoot ratio increased with forest age for fir (Fig. 7a), while it decreased with forest age for both spruce and pine (Fig. 7b,c). This study found that the root/shoot ratio was not always negatively correlated with forest stand age^51^. Therefore, researchers should be cautiously when using vegetation-specific root/shoot ratios to predict root biomass to avoid underestimation. The belowground competition for nutrients intensifies differently as various forests age, and the increased nitrogen deposition effect on the carbon allocation varies among forest types^15^. The mean annual temperature generally had a negative influence on the root/shoot ratio, while the mean annual precipitation had no consistent effect on the different forest types^52^. Therefore, the variation of the root/shoot ratio was due not only to environmental factors but also the specific species.

### Carbon stock potential and carbon sink

Using forest inventory data, a net global forest sink of 4.0 Pg C per year occurred over the past 20 years^5^. The old forest still plays an important role as a carbon sink^12^. Based on the past six forest inventory periods in China, old forests contributed 54.4% to the total carbon sink from 1977 to 2008^53^. In Tibet, the forest is mainly composed of middle-aged, mature and over-mature trees (Fig. 3; Table 1); however, it continues to function as a carbon sink (Table 3). The reason why carbon density of over-mature forest still increased can explained by the definition of over-mature forests. Coniferous forest with forest age more than 160a were all classified as over-mature forests, and even more than 80a were the over-mature forests for other forest types. However, the age threshold of carbon sink for forest can reach to 450–500a^17^. The forest age of Tibet were far from this threshold age (Fig. 3), and the carbon density kept increasing even for over-mature forest in Tibet^54^. On the other hand, we did not consider the influence of fire and deforestation on the suddenly decrease of carbon density. Other environmental factors, such as elevated CO_2_ concentration, nitrogen deposition and climate warming, can also stimulate the carbon sink of old forest^45^,^46^. These effects were all included in carbon density-age equations, because the equations were based on the plot data of primary forests, the long term growth of forests were all influenced by environmental factors. Although the carbon stock gradually increased from 2010 to 2050, the spatial distribution of the carbon stock variation was heterogeneous (Fig. 5). In China, temperate broadleaved deciduous forests were the largest carbon sinks, while cold temperate needle-leaved forest acted as a carbon source^53^. In Tibet, broadleaved mixed forests were generally a smaller carbon sink, while spruce, fir, pine and oak forests were larger carbon sinks (Fig. 6). Other forest types acted as smaller carbon sinks. However, these forest areas only accounted for 19.1% of the total in Tibet.

Hu *et al*.^23^ postulated that the carbon sink was 0.29 t C ha^−1^ yr^−1^ between 2005 and 2010 and would reach 0.28 t C ha^−1^ yr^−1^ between 2040 and 2050 in China^23^. The carbon sink was 0.36 C ha^−1^ yr^−1^ and 0.19 t C ha^−1^ yr^−1^, respectively, in Tibet for those same periods. This illustrates that the natural carbon-dense forests continue to have a strong carbon sink potential in China and will play an important role in the carbon cycle in the future. This result is in contrast to a previous study, which showed that both the carbon stock and carbon density decreased from 2006 to 2010 compared to 2001–2005 in Tibet^19^. In their study, the carbon sink was negative with respect to the biomass carbon density; however, the absence of carbon-dense forests can result in high uncertainty. In addition, the national-scale correlation used in that study may be not appropriate for the provincial scale because the environmental conditions are different between northeast and southwest China, which influences the mean and potential maximum carbon density. During the 2000 s, the average carbon sink in Tibet estimated here (0.36 Mg C ha^−1^ between 2001 and 2010) was lower than that in tropical forests (0.63 Mg C ha^−1^ between 1968 and 2007)^55^, in the United States (0.6 Mg C ha^−1^)^5^ and in Europe (1.3 Mg C ha^−1^)^5^; however, it continued to act as a net carbon sink.

**Figure 8:**
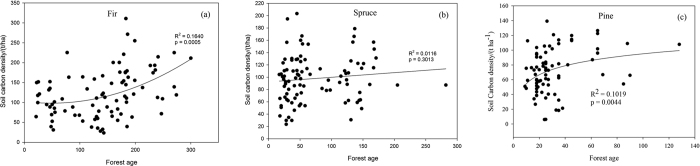
The relationship between soil carbon density in the top 50 cm and forest age for Fir (**a**), Spruce (**b**) and Pine (**c**) respectively in Tibet.

Carbon stocks continue to accumulate in live biomass as the forest ages^16^,^55^; however, the soil may not be a significant carbon sink, as indicated in other studies^14^. There was only a minor increase in the soil carbon density for fir and pine forests with age (Fig. 8a,c), and no significant increase was observed for the spruce forests in Tibet (Fig. 8b). Previous studies also showed that although aboveground tree biomass increased with forest age, mineral soil carbon was age-independent^56^,^57^ and organic soil carbon increased with age^56^. Although forest biomass increased significantly with forest age, the soil carbon stock may be more correlated with other factors, such as the soil texture and decomposition of soil organic matter^58^,^59^. Root-derived organic matter is more stable than shoot-derived organic matter and forms the majority of soil carbon^60^,^61^. Clear cutting and intensive logging not only release carbon sequestrated in the biomass but also decrease the soil carbon stock in organic and mineral soil via reduced inputs and disturbances^62^,^63^,^64^. The ratio of heterotrophic respiration to total soil respiration increases from 20% to 40% in mature forests to approximately 70–95% in young regenerating forests after clear cutting^65^,^66^. Therefore, the transformation from primary forests to planted forest may reduce soil carbon storage.

### Forest management for high carbon stock and carbon-dense forests

In general, the forests in Tibet are primary forests and are protected forests based on the Food and Agricultural Organization (FAO) definition^27^. Although the carbon sink is 4.0 Pg C yr^−1^ for global forests, deforestation emissions are 2.9 Pg C yr^−1 5^. Therefore, it is increasingly urgent to protect carbon-dense forests and old-growth forests from deforestation and degradation to avoid significant carbon emissions into the atmosphere^12^,^16^,^67^. Intensively managed forests can grow timber more efficiently, while primary forests tend to stock more carbon^11^. More of the assimilated carbon is allocated to aboveground pools in managed forests than in unmanaged forests^66^. A balanced budget of carbon was found based on the net primary production (NPP) dataset, while a soil carbon deficit was found based on the ratio of heterotrophic respiration to total detritus flux^66^. Carbon production (the ratio of NPP:GPP) decreases with increasing biomass and stand age^68^.

Elevated CO_2_ concentration, nitrogen deposition and climate warming may be harmful to the environment. However, these factors can stimulate tree growth, therefore enhancing forest C sequestration, especially elevated CO_2_ concentrations^46^. The current CO_2_ concentrations can still stimulate photosynthesis of tree species^69^. Based on the IPCC (2013) report, the CO_2_ concentration in the air will continue to increase through the end of the 21^st^ century^7^. The results of the modeled and field-warming experiments show a positive correlation between the mean annual temperature and NPP in boreal and tropical regions and a negative relationship for the alpine ecosystems on the Tibetan Plateau^70^. Therefore, the carbon sink capacity of forests may not be limited by climate change; rather, it is promoted by climate change.

The cessation of logging can prevent the release of carbon emissions to the atmosphere, and the continued growth of forests can assimilate additional carbon dioxide from the atmosphere^71^. Logging old-growth and young primary forests were equivalent for long-term emissions^67^. However, the carbon sink capacity of mature forests decreases with the forest stand age (Table 3). Planted and young forests will continue to absorb carbon dioxide more efficiently until 2050 in China^23^. Therefore, afforestation and reforestation remain efficient methods to maintain both the carbon amount and annual rate of carbon uptake. The choice of planted forest type is also very important. Fir and spruce forests have a larger carbon sink potential than other forest types in Tibet (Fig. 6). Therefore, the planted forests should use these forest types. Reducing human disturbance, forest fires and any other influences on the carbon pool in carbon-dense forests to avoid large carbon emissions is also a challenge. There is a large gap between the benefits of carbon storage and wood production. A planted forest can gain 5.18–6.05 Tg C (2326 km^2^) versus a carbon loss of 5.56 Tg C in logging areas (824 km^2^)^71^. Long harvest intervals are also needed to reduce carbon emissions for both forest management and timber usage^67^. More attention should be focused on both natural and anthropogenic disturbances in carbon-dense forests^16^.

## Conclusions

Precise estimation of the forest carbon stock and prediction of the carbon potential at the regional or global scale are significant research issues. In this study, empirical statistical equations of the carbon density and forest stand age for different forest types were developed to estimate and predict the carbon stock and carbon potential in Tibet. The results showed that Tibet’s total forest biomass carbon stock was 866.8 Tg C in 2010, with an average carbon density of 103.4 Mg C ha^−1^. The carbon stock and carbon density will increase to 969.4 Tg C and 115.6 Mg C ha^−1^, respectively, in 2050; however, the carbon sink capacity will decrease annually.

Our results provide regional-scale evidence that carbon-dense forests in Tibet will continue to act as a significant carbon sink in the next few decades. Fir, spruce and pine have larger annual carbon sink efficiencies, while fir, spruce and mixed broadleaved forests are the primary contributors to the carbon sink capacity in Tibet considering the influence of the carbon sink and the forest area. The carbon density increases more in young and middle-aged forests than in mature and over-mature forests, indicating that afforestation and reforestation will be an important method to maintain the role of carbon sinks in carbon-dense forests in Tibet. The planted fir and spruce forests will have larger carbon sink potentials than other forest types in Tibet. The spatiotemporal patterns of the forest carbon density documented in this study also benefit our understanding of the forest ecosystem carbon cycle and the management of forests in Tibet.

## Additional Information

**How to cite this article**: Sun, X.Y. *et al*. Forest biomass carbon stocks and variation in Tibet’s carbon-dense forests from 2001 to 2050. *Sci. Rep.* **6**, 34687; doi: 10.1038/srep34687 (2016).

## Supplementary Material

Supplementary Information

## Figures and Tables

**Table 1:** Proportions (%) of stage-classified forest area for each forest type in Tibet (Data were from the National forest inventory data in 2011).

Forest type	Young	Middle-age	Per-mature	Mature	Over-mature
Spruce	0.6	2.7	8.4	38.5	49.8
Fir	5.4	8.1	18.3	49.4	18.8
Larch	0.0	20.0	20.0	20.0	40.0
Sikang Pine	3.7	5.6	27.5	49.0	14.3
Yunnan Pine	1.0	6.1	20.3	55.8	16.8
Other pine	25.7	24.3	16.5	17.2	16.3
Oak	28.4	17.9	17.9	32.0	3.7
Birch	26.1	29.0	13.0	19.0	12.9
Poplar	29.9	32.7	18.8	18.7	0.0
Other hardwood	6.6	26.0	53.0	10.3	4.2
Coniferous mixed	21.3	0.6	16.5	20.5	41.2
Broadleaved mixed	7.4	31.0	11.6	14.7	35.2
Needle broad-leaved mixed	1.1	6.3	47.4	36.5	8.6

**Table 2:** The fitted carbon density-age equations for each forest type in Tibet.

Forest type	*a*	*b*	*c*	R^2^	n
Spruce*	165.4876	87.4437	42.8072	0.9244	52
Fir*	176.3957	69.7336	40.5151	0.9987	54
Larch*	109.2164	51.7521	29.5000	0.9878	26
Sikang pine†	118.5529	0.0234		0.6974	43
Yunnan pine*	84.6116	25.6789	21.9882	0.9888	37
Other pine*	122.0103	63.2502	36.7641	0.9975	28
Oak*	75.7111	153.4888	72.8788	0.7259	23
Birch*	160.9940	47.4269	20.3252	0.9657	27
Poplar†	36.1924	0.0327		0.8326	24
Other hardwood*	163.3400	38.9942	19.3798	0.9263	27
Coniferous mixed*	102.0122	66.7302	33.9307	0.8563	25
Broadleaved mixed*	110.6111	34.1375	17.5433	0.9823	34
Needle broad-leaved mixed†	87.7335	0.011		0.7338	27

Note: the carbon density-age equation was 
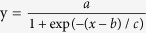
 for forest types marked with ^*^. The carbon density-age equation was y = a(1−exp(−bx)) for forest types marked with ^†^.

**Table 3:** Carbon density, carbon stock and carbon sink of Tibetan forests between 2001 and 2050.

Year	Carbon density (Mg C ha^−1^)	Carbon stock (Tg C)	Carbon sink (Tg C yr^−1^)
2001	99.1	831.1	
2010	103.4	866.8	3.6
2020	107.3	899.9	3.3
2030	110.7	928.0	2.8
2040	113.4	950.7	2.3
2050	115.6	969.4	1.9
