# Supplementary material for: Forest biomass carbon stocks and variation in Tibet’s carbon-dense forests from 2001 to 2050
Source: Sci Rep. 2016 Oct 5;6:34687. doi: 10.1038/srep34687 (PMC5050452; doi:10.1038/srep34687)
Supplement: Supplementary Information [file srep34687-s1.pdf]

Online Supplementary information

Forest biomass carbon stocks and variation in Tibet's carbon-dense forests from 2001 to 2050

Sun Xiangyang[1] Wang Genxu[1*] Huang Mei[2] Chang Ruiying[1] Ran Fei[1]

1. Institute of Mountain Hazards and Environment, Chinese Academy of Sciences, Chengdu Sichuan 610041
2. Institute of Geographic Sciences and Natural Resources Research, Chinese Academy of Sciences, Beijing 100101

***Corresponding author:**

Wang Genxu

Professor

Institute of Mountain Hazards and Environment, Chinese Academy of Sciences, #.9, Block 4, Renminnanlu Road, Chengdu Sichuan 610041,

Tel: +86-28-85233420

E-mail: wanggx@imde.ac.cn

This file contains Fig. S1, Table S1 and Table S2

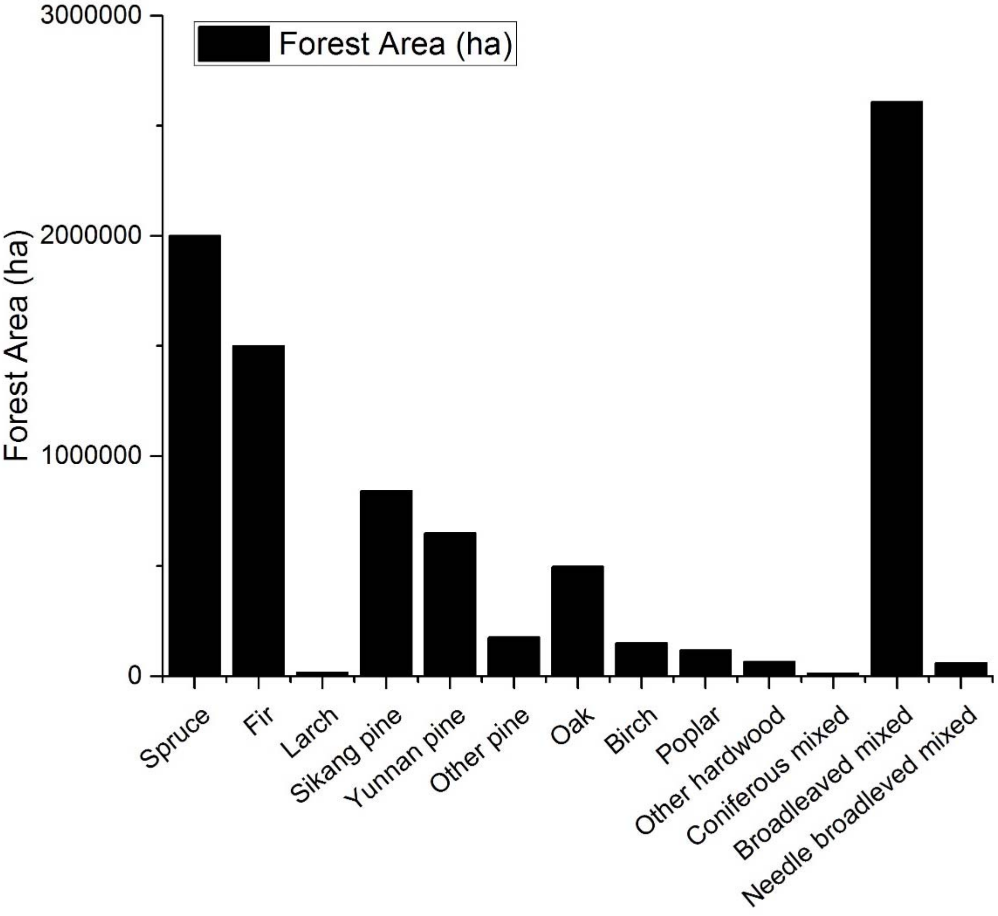

Fig. S1 The area of different forest types in Tibet

Table S1 Species-specific allometric equations of biomass components (B, Kg) to diameter at breast height (DBH, cm)

Tree species	Component	Coefficient symbol	Coefficient value	R^2	n
Spruce	Foliage	c	-2.994	0.803	125
		α	1.949		
	Branch	c	-3.308	0.910	
		α	2.231		
	Stem	c	-2.999	0.970	
		α	2.399		
	Root	c	-3.659	0.945	
		α	2.319		
Fir	Foliage	c	-4.059	0.929	103
		α	2.123		
	Branch	c	-4.108	0.949	
		α	2.348		
	Stem	c	-3.156	0.984	
		α	2.470		
	Root	c	-4.443	0.982	
		α	2.573		
Larch	Foliage	c	-3.708	0.846	68
		α	2.083		
	Branch	c	-3.963	0.915	
		α	2.369		
	Stem	c	-3.223	0.947	
		α	2.548		
	Root	c	-4.287	0.923	
		α	2.623		
Sikang pine	Foliage	c	-4.482	0.829	89
		α	2.254		
	Branch	c	-6.074	0.922	
		α	3.128		
	Stem	c	-2.136	0.993	
		α	2.535		
	Root	c	-3.135	0.960	
		α	2.447		
Yunnan pine	Foliage	c	-4.591	0.911	92
		α	2.183		
	Branch	c	-4.801	0.971	
		α	2.519		
	Stem	c	-3.017	0.915	
		α	2.398		
	Root	c	-4.33	0.939	
		α	2.381		

Other pine	Foliage	c	-0.648	0.996	68
		α	1.869		
	Branch	c	-3.561	0.998	
		α	2.206		
	Stem	c	-3.547	0.996	
		α	1.774		
	Root	c	-5.259	0.997	
		α	2.783		
Oak	Foliage	c	-1.805	0.980	65
		α	2.657		
	Branch	c	-3.009	0.878	
		α	1.823		
	Stem	c	-7.002	0.941	
		α	3.417		
	Root	c	-2.516	0.957	
		α	2.573		
Birch	Foliage	c	-1.035	0.963	53
		α	1.764		
	Branch	c	-3.623	0.963	
		α	2.494		
	Stem	c	-3.161	0.963	
		α	1.972		
	Root	c	-2.227	0.964	
		α	1.955		
Poplar	Foliage	c	-5.116	0.971	52
		α	2.341		
	Branch	c	-4.141	0.985	
		α	2.408		
	Stem	c	-2.627	0.976	
		α	2.312		
	Root	c	-3.605	0.99	
		α	2.333		
Other hardwood	Foliage	c	-4.780	0.989	73
		α	2.401		
	Branch	c	-4.699	0.958	
		α	2.808		
	Stem	c	-3.115	0.989	
		α	2.381		
	Root	c	-3.927	0.978	
		α	2.469		
Coniferous mixed	Foliage	c	-3.708	0.846	89
		α	2.083		
	Branch	c	-3.963	0.915	
		α	2.369		

	Stem	c	-3.223	0.947	
		α	2.548		
	Root	c	-4.287	0.923	
		α	2.623		
Broadleaved mixed	Foliage	c	-4.780	0.989	72
		α	2.401		
	Branch	c	-4.699	0.958	
		α	2.808		
	Stem	c	-3.115	0.989	
		α	2.381		
	Root	c	-3.927	0.978	
		α	2.469		
Needle broadleaved mixed	Foliage	c	-4.358	0.681	63
		α	2.530		
	Branch	c	-4.976	0.714	
		α	3.112		
	Stem	c	-3.411	0.812	
		α	2.780		
	Root	c	-3.257	0.612	
		α	2.063		

Note: Equations are of the form $\ln(B)=c+\alpha\ln(DBH)$, where B is biomass component and DBH is the diameter at breast height. The equation for larch is the same as coniferous mixed forest. The equation for other hardwood is the same as broadleaved mixed.

Table S2 Mean carbon content ratio for each parts of major tree species in Tibet

Trees	Component	Mean carbon content ratio	Trees	Component	Mean carbon content ratio
Spruce	Foliage	0.492	Fir	Foliage	0.522
	Branch	0.514		Branch	0.491
	Stem	0.443		Stem	0.468
	Root	0.481		Root	0.480
Larch	Foliage	0.508	Sikang pine	Foliage	0.480
	Branch	0.510		Branch	0.517
	Stem	0.488		Stem	0.465
	Root	0.490		Root	0.477
Yunnan pine	Foliage	0.508	Other pine	Foliage	0.493
	Branch	0.510		Branch	0.514
	Stem	0.487		Stem	0.476
	Root	0.490		Root	0.483
Oak	Foliage	0.439	Birch	Foliage	0.440
	Branch	0.460		Branch	0.486
	Stem	0.497		Stem	0.507
	Root	0.444		Root	0.483
Poplar	Foliage	0.438	Other hardwood	Foliage	0.440
	Branch	0.450		Branch	0.467
	Stem	0.428		Stem	0.500
	Root	0.442		Root	0.448
Coniferous mixed	Foliage	0.502	Broadleaved mixed	Foliage	0.445
	Branch	0.503		Branch	0.517
	Stem	0.458		Stem	0.4874
	Root	0.482		Root	0.477
Needle broadleaved mixed	Foliage	0.449			
	Branch	0.495			
	Stem	0.485			
	Root	0.465			
